# Effects of CO_3_^2−^ and OH^−^ on the solubility, metastable zone width and nucleation kinetics of borax decahydrate

**DOI:** 10.1098/rsos.181862

**Published:** 2019-06-26

**Authors:** Jing Chen, Jiaoyu Peng, Xingpeng Wang, Yaping Dong, Wu Li

**Affiliations:** 1 Key Laboratory of Comprehensive and Highly Efficient Utilization of Salt Lake Resources, Qinghai Institute of Salt Lakes, Chinese Academy of Sciences, 810008 Xining, Qinghai, People's Republic of China; 2 Engineering and Technology Research Center of Comprehensive Utilization of Salt Lake Resources, 810008 Xining, People's Republic of China; 3 University of Chinese Academy of Sciences, 100039 Beijing, People's Republic of China

**Keywords:** metastable zone width, solubility, nucleation kinetics, borax

## Abstract

Measurements of the solubility and metastable zone width (MZW) of borax decahydrate in sodium carbonate and sodium hydroxide aqueous were obtained. The onsets of nucleation were detected by the turbidity technique with the temperature range from 285 to 315 K. The results showed that the solubility of borax gradually decreased and the MZW broadened with the mass percentage of sodium carbonate increasing from 0% up to 9.22%. Correspondingly, the solubility and MZW had the same trend with the addition of sodium hydroxide. Meanwhile, the nucleation parameters of borax were determined and analysed to explain the trends obtained. Applying the classical three-dimensional nucleation theory approach, it was found that the addition of carbonate and hydroxide ions led to the values of solid–liquid interfacial energy (*γ*) increasing, which indicated the CO_3_^2−^ and OH^−^ ions adsorbed on the nuclei but suppressed nucleation rate.

## 1. Introduction

Boron compounds have unique advantages in their porosity, density and thermal stability, leading to potential applications for hydrogen storage, filtration, catalysis and optoelectronics [1–3]. In geological formations, boron compounds have different kinds of existing forms, such as sassoline, borax, ulexite and colemanite [1]. There is a growing interest in the crystallization of liquid and solid boron due to their long-term exploitation [4]. Brine is a major source of boron supply, and borax decahydrate (Na_2_B_4_O_7_·10H_2_O) is a typical crystalline product from brine [5]. For the investigation of crystallization of borax from brine, it is extremely important to know its solubility and MZW as functions of temperature and presence of other salts in the solution [6].

The major variables affecting the crystallization of borax from its solutions have been comprehensively investigated [7–9]. However, to obtain borax products from brine, evaporation processes are followed, which mainly depend on the species and quantity of coexisting compounds present in the solution [10]. Gurbuz & Ozdemir [11] investigated the effects of Ca^2+^ and Mg^2+^ by ultrasonic velocity technique. They found that trace amount of Ca^2+^ and Mg^2+^ had a slight effect on MZW of borax, but increasing the concentration will result in a reasonable increase. Peng [12–14] have investigated the influence of KCl, LiCl and K_2_SO_4_ on solubility and MZW using a laser technique. The results showed that KCl, LiCl and K_2_SO_4_ had a salt-in effect on borax. The MZW broadened with increasing the LiCl and K_2_SO_4_, but decreasing by KCl. The opposite effects of K_2_SO_4_ and KCl were attributed to the different mechanisms of Cl^−^ and SO_4_^2−^, suggesting that the effects of anions cannot be neglected.

Generally, there are several anions in brine, such as Cl^−^, SO_4_^2−^, CO_3_^2−^ and OH^−^ [15]. CO_3_^2−^ ion is one of the main components of carbonate-type brine which always contains a large amount of boron. A typical example is Zabuye salt lake [16]. Different concentrations of CO_3_^2−^ lead to different pH values, thus affecting the solubility and MZW of borax in brine. Although there are several reports on the solubility and MZW of borax, the results are still inadequate and roughly compared with cations. Therefore, the aim of this study was to investigate the influence of CO_3_^2−^ and pH on solubility and the MZW of borax using the polythermal method. Then the experimental MZW data of different cooling/heating rates *R* were analysed and discussed by three-dimensional nucleation theory.

## 2. Experiment section

### 2.1. Material and methods

All of the chemical reagents used in this study are listed in table 1. Na_2_B_4_O_7_·10H_2_O was recrystallized with a purity more than 99.99%. Water (resistivity, 18.25 MΩ cm^−1^) was deionized from a water purification system (UPT-II-20T, Chengdu Ultrapure Technology Co., Ltd) before experiments.

**Table 1.** Chemical reagents employed in the experiment.

chemical name	formula	provider	purity
borax decahydrate	Na_2_B_4_O_7_·10H_2_O	Tianjin Damao Chemical Reagent Factory	≥99.99%
sodium carbonate anhydrous	Na_2_CO_3_	Tianjin Yongda Chemical Reagent Development Center	≥99.95%
sodium hydroxide	NaOH	Tianjin Kermel Chemical Reagent Development Center	≥99.95%

**Figure 1.**
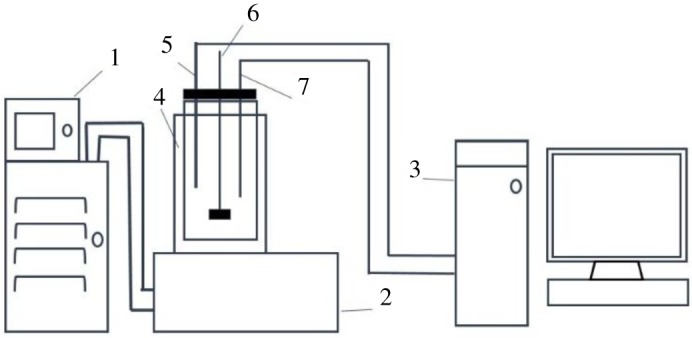
Schematic representation of experimental set-up. (1, low constant temperature bath; 2, temperature control system; 3, a computer processing system; 4, crystallizer; 5, turbidity sensor; 6, overhead stirring; 7, temperature sensor.)

The experimental set-up is shown in figure 1. A turbidity meter was employed to detect nucleation/dissolution. The temperature of prepared solution was measured using the digital thermometer with precision of ±0.1°C. Cooling rates control was accomplished using a Crystal SCAN with four parallel reactors (E1061, HEL, UK) containing systems for temperature control and computer processing as well as a crystallizer assisted with programmable thermostatic bath (FP50-ME, Julabo, Germany). The crystallizer was a 100 ml glass vessel with an internal overhead stirrer, temperature sensor and turbidity sensor. Besides, the crystallizer was made air-tight so that the loss of solvent due to evaporation could be minimized. The X-ray diffraction (XRD) analysis (X'Pert PRO, 2006 PANalytical) was used to confirm the identity of the solid phase crystallizing from the solutions. The pH was measured by pH meter (S470 Seven Excellence, Mettler Toledo) with precision of ±0.05.

### 2.2. Solubility and MZW determination

The determination of the solubility and MZW of the borax in sodium carbonate solutions was carried out with a temperature range from 285 to 315 K according to the conventional polythermal method. Firstly, 60 g of mixture was placed into a 100 ml crystallizer. Then, the mixture was heated with a given rate above the saturation temperature for 10 min to ensure complete dissolution of the solid phase. Finally, the solution was cooled down with the same constant rate until the first visible nucleus appeared, which can be detected by a sudden increase in turbidity. The corresponding temperature at the point of nucleation and dissolution was recorded as *T*_1_ and *T*_2,_ respectively. The above steps were repeated at five cooling/heating rates of 55, 45, 35, 25 and 15 K h^−1^ and at constant impeller speed of 300 r.p.m. The concentration of borax and sodium carbonate in the solution was obtained by titration. The faster the heating rates were, the higher the measured dissolution temperature was. Therefore, the saturation temperature *T*_0_ of borax can be obtained by extrapolating the *T*_2_–*R* curve to a virtual heating rate of ‘zero’. The metastable zone width (MZW) of borax is represented by the maximum undercooling Δ*T*_max_ (Δ*T*_max_ = *T*_0_ − *T*_1_).

### 2.3. Chemical analysis

The boron content was determined by mannitol conversion acid−base titration. The CO_3_^2−^ ion concentration was determined by adding 0.05 mol l^−1^ HCl, and using methyl red-bromcresol green as indicator. The accuracy of these analyses was about 0.1%. All of the estimated uncertainties of the research are listed in table 2.

**Table 2.** Uncertainties of measurements estimated for this research.

property	estimated uncertainty
solubility	±0.05 g of 100 g of H_2_O
temperature	±0.06°C
pH	±0.03
*w* %	±0.08

## 3. Results and discussion

### 3.1. XRD analysis

**Figure 2.**
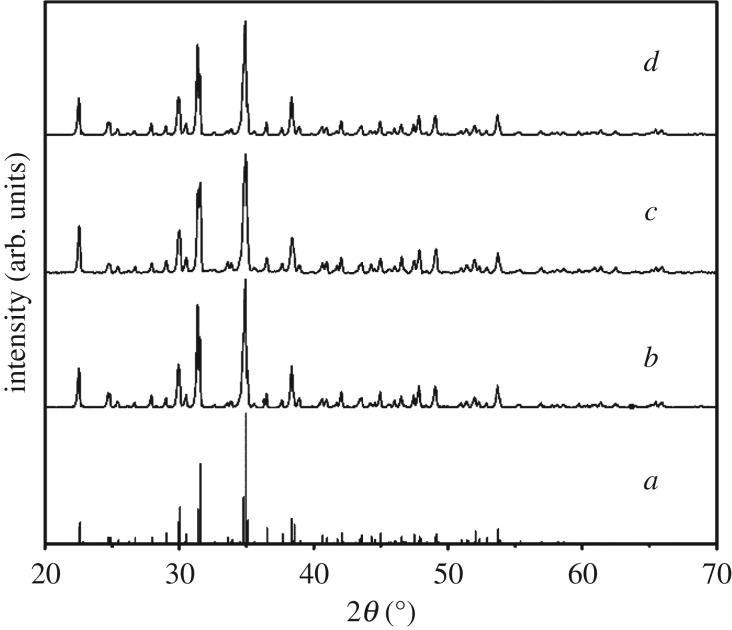
The XRD patterns of crystallized borax decahydrate: (*a*) standard; (*b*) crystallized in pure water; (*c*) crystallized in sodium hydroxide solution; (*d*) crystallized in sodium carbonate solution.

The XRD patterns of borax obtained from pure water, sodium hydroxide and sodium carbonate were investigated, respectively, shown in figure 2. It manifests the XRD patterns are identical and well indexed to borax without any impurities, according to the reference data JCPDS 75-1078.

### 3.2. Solubility

**Figure 3.**
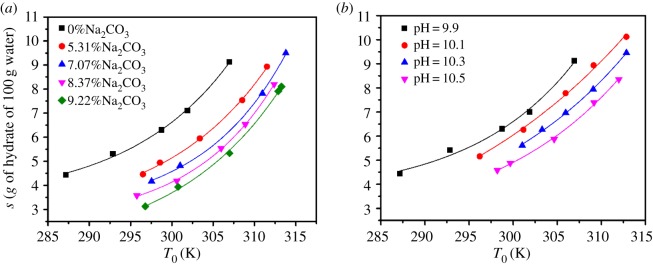
Solubility of borax (*a*) effects by CO_3_^2−^; (*b*) effects of pH adjusted by NaOH.

The solubility of borax in different mass percentages of sodium carbonate (0.0–9.22%) in aqueous solution was determined. The obtained experimental solubility data are demonstrated graphically in figure 3. It can be observed in figure 3*a* that the solubility of borax increased with temperature, which can be attributed to the thermal motion of molecule. Besides, the addition of sodium carbonate leads to the solubility decrease. It could be the common ion effect that the addition of sodium carbonate releases Na^+^ into the solution, making dissolution−precipitate equilibrium move towards the precipitate. Therefore, the solubility of borax decreases. It also can be seen that the solubility curves are roughly paralleled to each other, indicating that the increment of sodium carbonate causes a gradual decrement of borax solubility.

Furthermore, it can be seen that pH increases with the addition of Na_2_CO_3_ (from 0 to 9.22%), based on figure 3*a*. Therefore, pH range from 9.9 to 10.5 was selected to investigate by the addition of Na_2_CO_3_ and NaOH, respectively. As shown in figure 3*a*, the solubility of borax at Na_2_CO_3_ concentration of 9.22% (pH = 10.5) was lower than pH = 10.5 adjusted by NaOH, seen in figure 3*b*. It suggests that CO_3_^2−^ has more prominent effects on the decrement of borax solubility than OH^−^.

### 3.3. Thermodynamic properties of borax

Dissolution enthalpy, Δ_dis_*H*, and dissolution entropy, Δ_dis_*S*, are important to investigate the dissolution behaviour of the solute in different solvents. When the solubility of borax in sodium carbonate and sodium hydroxide solution at different temperatures is available, then the values of Δ_dis_*H* and Δ_dis_*S* can be determined from the van't Hoff equation as follows:

3.1 $$\ln x=\frac{-\Delta_{\mathrm{dis}}H}{R_{G}T_{0}}+\frac{\Delta_{\mathrm{dis}}S}{R_{G}},$$

**Figure 4.**
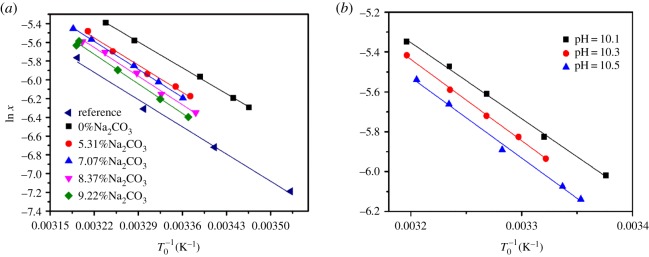
The van't Hoff plots of ln *x* versus 1/*T*_0_. (*a*) In sodium carbonate solution; (*b*) in sodium hydroxide solution.

Where Δ_dis_*H* and Δ_dis_*S* are the dissolution enthalpy and entropy, respectively, *R*_G_ is gas constant (8.314 J mol^−1^ K^−1^) and *x* is the mole fraction of borax. The van't Hoff plots shown in figure 4 are obtained from the linear fit of ln *x* versus 1/*T*_0_. Then the dissolution enthalpy and entropy of borax which are shown in table 3 can be calculated from the slope and the interception of these plots.

**Table 3.** Dissolution enthalpy and entropy of borax in sodium carbonate solutions.

factor	value	Δ_dis_*H* (kJ mol^−1^)	Δ_dis_*S* (J mol^−1^ K^−1^)	*R*^2^
*w* % (Na_2_CO_3_)	0.00 [17]	34.11	61.14	0.9909
	0.00	32.84	61.51	0.9914
	5.31	34.12	64.21	0.9908
	7.07	36.66	71.80	0.9987
	8.37	37.37	73.27	0.9694
	9.22	37.99	74.38	0.9954
pH	10.1	31.72	57.01	0.9977
	10.3	33.62	61.62	0.9985
	10.5	33.92	62.33	0.9942

The values of dissolution enthalpy and entropy of borax are 34.11 kJ mol^−1^ and 61.14 J mol^−1^ K^−1^ [17] in the literature, which are in good agreement with our experimental data. It can be found from table 3 that the dissolution enthalpy and entropy are positive, which indicates that the dissolution is always endothermic and entropy driven. It also can be found that the mixture with more sodium carbonate and sodium hydroxide has lower solubility but higher values of Δ_dis_*H* and Δ_dis_*S*, which is consistent with general thermodynamic principles [18].

### 3.4. Metastable zone width

**Figure 5.**
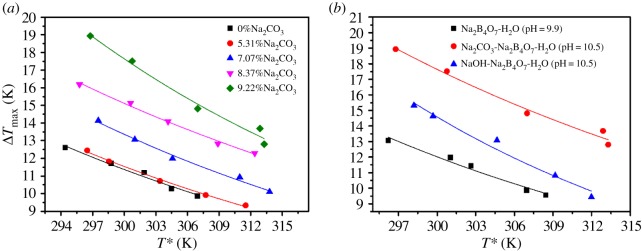
Changes in MZW (*R* = 55 K/h): (*a*) at different mass percentages of sodium carbonate solutions; (*b*) at same pH.

The MZW data of borax against saturation temperature at different mass percentages of sodium carbonate are given in figure 5*a*. It is clear that the MZW becomes broad with the increase of sodium carbonate. The effects are more significant at higher mass percentages, but have little effect with mass percentages of sodium carbonate below 5.31%. The effects could have two possible explanations. One is that carbonate ions with large size may block the active growth sites of the nuclei forming in bulk solution due to steric effect, which might depend on concentrations. When presented in relatively small concentrations, these ions suppress nucleation slightly. Therefore, it might be seen that the higher mass percentages of carbonate ions, the more powerful the inhibiting effects are. Another possible reason is that carbonate ions might act as surface active agents, rendering the nuclei inactive [19].

The results of the influences of pH on MZW are also given in figure 5*b*. It should be noted that the MZW of borax is larger at higher pH. This could be explained by the relationship between the pH and polyborate speciation [2]. Based on the reports [20,21], the speciation of boron strongly depends on the chemical medium, especially the pH. It appears that tetraborate species exist in solution for pH ranging from 7 to 12 and are abundant at about pH = 10. With the higher pH, the concentration of B_4_O_5_(OH)_4_^2−^ will decrease, which makes nucleation more difficult. In consequence, the MZW is broadened. Besides, when the solution pH remains at 10.5, seen in figure 5*b*, it can be easily found that the MZW of borax in the Na_2_CO_3_–NaB_4_O_7_–H_2_O system is wider than that of the NaOH–NaB_4_O_7_–H_2_O system. It may be the impurities in the former system are CO_3_^2−^ and OH^−^. Nevertheless, there is only OH^−^ ion in the NaOH–NaB_4_O_7_–H_2_O system. The extra CO_3_^2−^ ion in sodium carbonate solution would retard nucleation so that the MZW is broadened.

### 3.5. Classical three-dimensional nucleation theory approach

**Figure 6.**
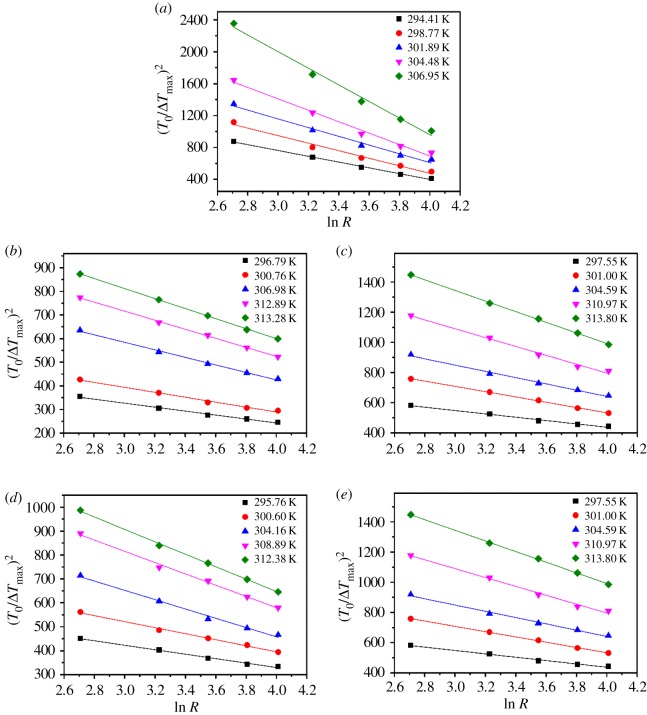
The plot of (*T*_0_*/*Δ*T*_max_)^2^ versus ln *R* for borax in different mass percentages of sodium carbonate: (*a*) 0.00% Na_2_CO_3_; (*b*) 5.31% Na_2_CO_3_; (*c*) 7.09% Na_2_CO_3_ ; (*d*) 8.38% Na_2_CO_3_; (*e*) 9.22% Na_2_CO_3._

**Figure 7.**
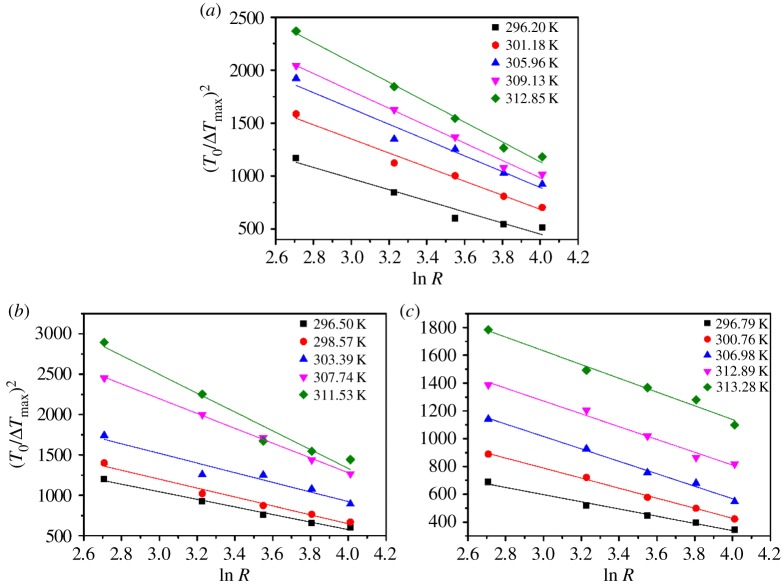
The plot of (*T*_0_*/*Δ*T*_max_)^2^ versus ln *R* for borax at: (*a*) pH = 10.1; (*b*) pH = 10.3 ; (*c*) pH = 10.5_._

The solid–liquid interfacial energy *γ* is an important thermodynamic parameter that indicates the ability of the solute to crystallize from solution [22]. According to the classical three-dimensional nucleation theory, the relationship between MZW and cooling/heating rate *R* can be represented as equation (3.2) [23,24]. Where *k*_B_ is the Boltzmann's constant equal to *R*_G_/*N*_A_ (*N*_A_ is the Avogadro number); where *A* is a kinetic constant associated with the media of nucleation; *f* is a constant expressing the number of nuclei in certain volume; Ω is the molecular volume, calculated by the density. Figures 6 and 7 present plots of (*T*_0_/Δ*T*_max_)^2^ against ln *R* for borax in different percentage fractions of sodium carbonate and sodium hydroxide solutions according to equation (3.2). The values of *γ* and *A* can be calculated from the slope and the intercept by equations (3.3)–(3.5), respectively

3.2 $${\left(\frac{\Delta T_{\max}}{T_{0}}\right)}^{2}=F-F_{1}\ln R=F(1-Z\ln R),$$

where *F*, *Z* and *B* represent slope, intercept and nucleation parameter, values are calculated by equations

3.3 $$F=\frac{1}{ZB}{\left(\frac{\Delta_{\mathrm{dis}}H}{R_{G}T_{\lim}}\right)}^{2},$$

3.4 $$Z=\frac{F_{1}}{F}=\ln\left(\frac{f}{AT_{0}}\frac{\Delta H_{s}}{R_{G}T_{\lim}}\right)$$

3.5 $$\mathrm{and}\;\;B=\frac{16\pi }{3}{\left(\frac{\gamma \Omega^{1/3}}{k_{B}T_{\lim}}\right)}^{3}.$$

As shown in table 4, the estimated solid–liquid interfacial energy *γ* in pure water is 1.81 mJ^−3^ m^−2^, which agrees well with the data 1.7 in the previously published literature [25]. It should be noted that the values of solid–liquid interfacial energy *γ* decrease with an increase in saturation temperature *T*_0_, but increase with the addition of Na_2_CO_3_, seen from table 4. Based on the reported articles, it is known that the increase in the value *γ* will suppress nucleation rate and broaden the MZW. Basically, the higher interfacial energy means the bigger nuclear barrier which leads to the harder nucleation process. According to the data from table 4, it could be concluded that the adsorption of CO_3_^2−^ on the nucleus surface leads to the increase in *γ* [26]. Furthermore, the solid–liquid interfacial energy *γ* was less in NaOH–NaB_4_O_7_–H_2_O system than that of Na_2_CO_3_–NaB_4_O_7_–H_2_O system, seen from table 5. It can be explained by the fact that the charge of OH^−^ is less than CO_3_^2−^ so that the adsorption is weaker.

**Table 4.** Values of kinetic parameters of borax at different mass percentages of sodium carbonate estimated using classical three-dimensional nucleation theory (units: *T*_0_, K; *γ*, mJ^−3^ m^−2^; *A*, * *10^24^ m^−3^ h^−1^).

*w*% (Na_2_CO_3_)	*T*_0_	nucleation equation	*A*	*γ*	*R*^2^
0.00	294.41	(*T*_0_/Δ*T*_max_)^2^ = −362.37 ln *R* + 1849.18	2.80	2.52	0.9937
	298.77	(*T*_0_/Δ*T*_max_)^2^ = −472.07 ln *R* + 2364.52	2.90	2.31	0.9994
	301.89	(*T*_0_/Δ*T*_max_)^2^ = − 547.25 ln *R* + 2800.16	3.38	2.22	0.9933
	304.48	(*T*_0_/Δ*T*_max_)^2^ = −716.02 ln *R* + 3557.29	3.89	2.04	0.9953
	306.95	(*T*_0_/Δ*T*_max_)^2^ = −1041.34 ln *R* + 5125.39	4.64	1.81	0.9990
5.31	296.50	(*T*_0_/Δ*T*_max_)^2^ = −140.56 ln *R* + 1131.58	3.03	3.45	0.9966
	298.57	(*T*_0_/Δ*T*_max_)^2^ = −165.62 ln *R* + 1300.76	3.14	3.27	0.9996
	303.39	(*T*_0_/Δ*T*_max_)^2^ = −220.39 ln *R* + 1688.51	3.63	2.99	0.9963
	307.74	(*T*_0_/Δ*T*_max_)^2^ = −308.56 ln *R* + 202.60	4.16	2.69	0.9982
	311.53	(*T*_0_/Δ*T*_max_)^2^ = −354.87 ln *R* + 2546.63	4.97	2.55	0.9959
7.09	297.55	(*T*_0_/Δ*T*_max_)^2^ = −110.68 ln *R* + 880.04	2.79	3.80	0.9879
	301.00	(*T*_0_/Δ*T*_max_)^2^ = −174.97 ln *R* + 1232.95	3.32	3.28	0.9989
	304.59	(*T*_0_/Δ*T*_max_)^2^ = −207.83 ln *R* + 1472.73	3.94	3.12	0.9921
	310.97	(*T*_0_/Δ*T*_max_)^2^ = −293.85 ln *R* + 1971.71	4.86	2.80	0.9901
	313.53	(*T*_0_/Δ*T*_max_)^2^ = −352.01 ln *R* + 2400.10	5.28	2.64	0.9993
8.38	295.76	(*T*_0_/Δ*T*_max_)^2^ = −92.97 ln *R* + 701.67	2.51	4.08	0.9917
	300.60	(*T*_0_/Δ*T*_max_)^2^ = −125.94 ln *R* + 899.25	3.01	3.72	0.9940
	304.16	(*T*_0_/Δ*T*_max_)^2^ = −134.00 ln *R* + 1000.39	3.67	3.66	0.9858
	308.89	(*T*_0_/Δ*T*_max_)^2^ = −235.34 ln *R* + 1521.09	4.34	3.05	0.9936
	312.38	(*T*_0_/Δ*T*_max_)^2^ = −259.56 ln *R* + 1685.47	4.74	2.97	0.9982
9.22	296.79	(*T*_0_/Δ*T*_max_)^2^ = −84.17 ln *R* + 579.43	3.40	4.31	0.9902
	300.76	(*T*_0_/Δ*T*_max_)^2^ = −104.23 ln *R* + 706.29	3.79	4.04	0.9881
	306.98	(*T*_0_/Δ*T*_max_)^2^ = −159.14 ln *R* + 1061.86	4.04	3.54	0.9948
	312.89	(*T*_0_/Δ*T*_max_)^2^ = −191.60 ln *R* + 1291.13	5.25	3.35	0.9988
	313.28	(*T*_0_/Δ*T*_max_)^2^ = −212.14 ln *R* + 1448.67	5.40	3.24	0.9995

**Table 5.** Values of kinetic parameters of borax at different pH values estimated using classical three-dimensional nucleation theory (units: *T*_0_, K; *γ*, mJ^−3^ m^−2^; *A*, 10^24^ m^−3^ h^−1^).

pH	*T*_0_	nucleation equation	*A*	*γ*	*R*^2^
10.1	296.20	(*T*_0_/Δ*T*_max_)^2^= −534.51 ln *R* + 2550.10	10.47	2.16	0.9386
	301.18	(*T*_0_/Δ*T*_max_)^2^ = −665.23 ln *R* + 3346.68	10.13	2.01	0.9760
	305.96	(*T*_0_/Δ*T*_max_)^2^ = −745.82 ln *R* + 3875.80	9.84	1.95	0.9540
	309.13	(*T*_0_/Δ*T*_max_)^2^ = −820.24 ln *R* + 4264.34	9.63	1.89	0.9884
	312.85	(*T*_0_/Δ*T*_max_)^2^ = −938.72 ln *R* + 4889.24	9.39	1.82	0.9905
10.3	296.50	(*T*_0_/Δ*T*_max_)^2^ = −468.49 ln *R* + 2449.70	10.25	2.26	0.9862
	298.57	(*T*_0_/Δ*T*_max_)^2^ = −551.84 ln *R* + 2855.87	10.01	2.14	0.9754
	303.39	(*T*_0_/Δ*T*_max_)^2^ = −597.27 ln *R* + 3311.14	9.97	2.10	0.9188
	307.74	(*T*_0_/Δ*T*_max_)^2^ = −917.03 ln *R* + 4947.78	9.68	1.82	0.9983
	311.53	(*T*_0_/Δ*T*_max_)^2^ = −1164.65 ln *R* + 5991.67	9.35	1.69	0.9525
10.5	296.79	(*T*_0_/Δ*T*_max_)^2^ = −234.64 ln *R* + 1280.51	10.60	2.73	0.9850
	300.76	(*T*_0_/Δ*T*_max_)^2^ = −361.28 ln *R* + 1872.36	10.39	2.45	0.9964
	306.98	(*T*_0_/Δ*T*_max_)^2^ = −448.20 ln *R* + 2360.97	10.03	2.30	0.9918
	312.89	(*T*_0_/Δ*T*_max_)^2^ = −461.23 ln *R* + 2655.04	9.85	2.29	0.9814
	313.28	(*T*_0_/Δ*T*_max_)^2^ = −472.77 ln *R* + 3068.73	9.82	2.28	0.9140

## 4. Conclusion

The effects of sodium carbonate and sodium hydroxide on the solubility and MZW of borax have been studied at temperature ranging from 285 to 315 K using turbidity technique. A salting-out effect was observed under the larger mass percentages of the sodium carbonate and sodium hydroxide, which resulted in the lower solubility of borax. It was found that the addition of sodium carbonate broadened the MZW significantly, and the influence depended on concentration. It was believed that the addition of sodium carbonate adsorbed on nuclei and suppressed the activities of nuclei in the solution which enabled the larger MZW. In addition, the pH had an effect on polyborate species. The increasing of pH could make the concentration of the tetraborate decrease, which led to the broadening of MZW. Finally, the obtained MZW data were analysed with the classical three-dimensional nucleation theory approach. The value of solute–solvent interfacial energy *γ* increased as the mass percentage of sodium carbonate was larger. The investigation of these parameters is very useful for the design and development of a crystallization process.

## Supplementary Material

Reviewer comments

## Data accessibility

This article does not contain any additional data.
